# Cavitation-induced shock wave behaviour in different liquids

**DOI:** 10.1016/j.ultsonch.2023.106328

**Published:** 2023-02-14

**Authors:** Mohammad Khavari, Abhinav Priyadarshi, Justin Morton, Kyriakos Porfyrakis, Koulis Pericleous, Dmitry Eskin, Iakovos Tzanakis

**Affiliations:** a School of Computing and Engineering, College of Science and Engineering, University of Derby, Derby DE22 3AW, United Kingdom; b Faculty of Technology, Design and Environment, Oxford Brookes University, Oxford OX33 1HX, United Kingdom; c Faculty of Engineering and Science, University of Greenwich, Central Avenue, Chatham Maritime, Kent ME4 4TB, United Kingdom; d Computational Science and Engineering Group, University of Greenwich, 30 Park Row, London SE10 9LS, United Kingdom; e Brunel Centre for Advanced Solidification Technology, Brunel University London, Uxbridge UB8 3PH, United Kingdom; f Department of Materials, University of Oxford, Parks Rd, Oxford OX1 3PH, United Kingdom

**Keywords:** Ultrasonic cavitation, Shock wave, Bubble cloud

## Abstract

This paper follows our earlier work where a strong high frequency pressure peak has been observed as a consequence of the formation of shock waves due to the collapse of cavitation bubbles in water, excited by an ultrasonic source at 24 kHz. We study here the effects of liquid physical properties on the shock wave characteristics by replacing water as the medium successively with ethanol, glycerol and finally a 1:1 ethanol–water solution. The pressure frequency spectra obtained in our experiments (from more than 1.5 million cavitation collapsing events) show that the expected prominent shockwave pressure peak was barely detected for ethanol and glycerol, particularly at low input powers, but was consistently observed for the 1:1 ethanol–water solution as well as in water, with a slight shift in peak frequency for the solution. We also report two distinct features of shock waves in raising the frequency peak at MHz (inherent) and contributing to the raising of sub-harmonics (periodic). Empirically constructed acoustic pressure maps revealed significantly higher overall pressure amplitudes for the ethanol–water solution than for other liquids. Furthermore, a qualitative analysis revealed that mist-like patterns are developed in ethanol–water solution leading to higher pressures.

## Highlights

• Spatio-temporal evolution of shock wave (SW) dynamics studied in different liquids.
• Shifting of SW frequency peak attributed to changes in speed of sound (V).
• Cavitation zone and shielding increase with input power in all liquids but glycerol.
• Ethanol-water mixture deemed to be suitable for synthesis of nanomaterials.

## 1 Introduction

Shock waves from collapsing cavitation bubbles have been the subject of interest to academics and industrialists due to their extraordinary potential of generating severe response dynamics in a variety of fields, ranging from biomedical sciences, nanomaterials, food processing, microelectronics, to liquid metal processing and additive manufacturing [1], [2], [3], [4], [5], [6]. Also recently, fundamental studies investigated the spatial shock wave pressure in the cavitation centre and the threshold of shock waves upon solid–liquid impact respectively [7], [8] further advancing the existing knowledge in the field. Hence, understanding the acoustically-induced cavitation shock waves, and specifically the detection and elucidation of acoustic signals (that can be used as a powerful tool to control cavitation activity during ultrasonic processing as in [9], [10]) emanated from bubble activity is vital for the development and further improvement of various applications and techniques allied to these fields. Shock wave characterisation in sonicated liquids subsequent to the introduction of high intensity ultrasonic waves has long been scrutinised in a number of studies [11], [12], [13], [14], [15], [16], [17], [18]. Perhaps the most important feature of acoustic cavitation is the multiple shock wave formation and propagation due to micro-bubble and cloud collapses. These shock waves can propagate with huge velocities creating a powerful pressure field in the surrounding medium [19]. The precise characteristic evaluation of these emitted high-energy shock waves helps to underpin several aspects of physical, chemical and biological interventions, such as grain refinement in liquid metals [20], liquid exfoliation of nanomaterials [21], sonochemistry [22] and lithotripsy [23], to name but a few. Therefore, visualizing and quantifying these shock waves becomes essential for harnessing this remarkable energy and understanding its nature.

The identification of shock strength is often difficult in ultrasonic environments owing to large pressure fluctuations during cavitation activity and complex bubble interactions resulting from a non-linear acoustic response. To explore and meaningfully describe the shock wave characteristics in a complex multi-bubble liquid system, a systematic experimental approach is necessary. This requires the use of advanced sensors capable of detecting the cavitation activity and broadband acoustic emissions. With the advent of extremely sensitive high frequency cavitation sensors, measuring the shock wave pressure and strength has now become possible. Numerous studies have been conducted in the past that involve experimental pressure measurements in relation to shock wave propagation [13], [16], [24], [25], [26], [27], [28]. Johnston et al. [13] showed that the periodic shock waves emanating from the cloud of bubbles generated from transducers operating at 254 kHz were related to acoustically induced sub-harmonics (and higher order harmonic signals) and were principally governed by the sonication intensity. Khavari et al. [27] further reported that cavitation-induced shock waves in a water medium from a 24 kHz source, lead to a strong signal peak in the pressure spectrum, at around 3.2 MHz. A high order resolution of the cavitation spectra additionally allows for the accurate determination and quantification of the shock strength and distribution within the sonicated medium. The ultrasonically driven cavitating bubbles also act as secondary acoustic sources and the generated cavitation spectrum is a combination of the driving frequency and the intensity of primary acoustic signals in addition to the bubble field characteristics [29]. It should be noted here that in all the aforementioned shock wave characterisation studies, water-based systems were mostly used and therefore it still remains unclear how the cavitation dynamics and shock wave propagation occur in liquids with largely different physical properties.

Specifically, the strength of propagating shock waves in multi-bubble systems resulting from cavitation implosions is dependent primarily on the speed of sound in the liquid and interfacial properties such as surface tension, viscosity, gas content, vapour pressure and density. However, shock wave appearance in other ultrasonicated liquids that find potential use in applications such as graphite exfoliation [28], sonochemical synthesis [30], emulsification [31] etc. is very limited. Garen et al. [32] recently reported that in highly viscous liquids such as glycerol, collapse shocks from laser induced bubbles were absent and cavitation occurred through shockless rebounds at low temperatures. On the contrary, for distilled water the rebound event was always supplemented with collapse shocks as reported elsewhere [19], [20], [33]. Other studies on viscosity influence, - though not specifically conducted to analyse shock wave behaviour, did reflect upon the effect of liquid properties on the overall cavitation dynamics. For example, Žnidarčič et al. [34] studied the influence of liquid viscosity on ultrasonic cavitation and the turbulence intensity in glycerol and ethylene glycol. It was observed that there was no significant effect of viscosity on the main bubble dynamics of the cavitation structure attached to the horn tip, but did result in a substantial decrease in the nucleation of tiny cavitation bubble clouds leading to more intense shock negative pressure peaks close to the source owing to less cushioning by the non-condensed bubble cloud. Schaad [35] also studied the viscosity effects in the case of hydrodynamic cavitation by comparing water and glycol and found that cavitation is less pronounced in glycol than in water. Tzanakis et al. [6] recently investigated the acoustic cavitation effects in a highly viscous fibre impregnated thermoplastic melt (polylactide) and found that the active cavitation zone is highly restricted to only a couple of millimetres beneath the horn tip with strong attenuation and almost no shielding effects.

In this study, we aim to extend the work presented in [27] to other liquids having different physical properties in comparison to water. To this end, we have chosen a number of model systems with a wide range of density, surface tension and viscosity including ethanol, glycerol and 1:1 ethanol–water solution as well as water. A low frequency sonication source at 24 kHz generated numerous cavitation bubbles and bubbly clouds that upon collapse emitted powerful shock waves. Calibrated fibre-optic acoustic sensors in the vicinity of the cavitation region captured the propagating shock waves. The corresponding acoustic pressure spectrum was analysed and the effect of shock waves in the tested liquids was studied. Finally, we conducted a comprehensive parametric study for various transducer powers for the studied liquids, and for a wide range of sensor positions. Pressure distribution maps were reconstructed for each liquid analysing 55 different locations with more than 1.5 million individual cavitation collapsing events taken into account. To the best of our knowledge, no research has previously been performed in this context. The findings of this study will help scientists and engineers understand the shock wave mechanisms and their role in the aforementioned applications.

## 2 Methodology

The experimental setup is shown in Fig. A1. Four different liquids (ethanol, glycerol, 1:1 ethanol–water solution and water) with distinctly different physical and cavitation properties [27] were chosen for this analysis. Sonication was applied by a 24 kHz transducer (UP200S, Hielscher Ultrasonics GmbH) to the working liquid inside a glass tank (300 mm × 75 mm × 100 mm). Acoustic pressures were captured with a fibre-optic hydrophone FOH (Precision Acoustics, ltd.) calibrated over the range of 1–30 MHz at 1 MHz increments. Furthermore, a high-speed camera (SA-Z, Photron, ltd.) was used to capture the cavitation emissions (a second similar experimental setup described elsewhere [27], [28] was used to capture and record the shock wave propagation in different liquids from which some of our Supplementary Viddeos are recorded). We chose 55 positions (corresponding to more than 1300 experiments) across the liquid tank for acoustic pressure measurements for each of the studied liquids. All experiments for each position were performed, at least twice, for three different input transducer powers: 20 %, 60 % and 100 %, corresponding to peak-to-peak displacement amplitudes of 42, 126 and 210 µm, respectively. We chose the centre of the cylindrical Ti sonotrode tip (3 mm in diameter) as the origin and placed the probe at *x* = −10, −8, −5, −3, −1, 0, 1, 3, 5, 8 and 10 mm and *y* = 1, 3, 5, 8 and 10 mm to obtain a broad symmetric acoustic pressure map. However, due to chaotic nature of cavitation, pressure peaks weren’t seemingly distributed. There is a tendency for pressure peaks to appear mainly on the right-hand side of this map (for example see water/ethanol mixture with no pressure peaks at 10 mm on the left side). This is related with the direction of the acoustic streaming as previously seen and explained in [36] as well as with acoustic shielding [37]. Real-time acoustic signals for 60 waveforms were recorded by a digital oscilloscope PicoScope-3204D (Pico Technology) with a high sampling rate of 500 × 10^6^ samples/s under steady-state conditions [27]. The experimental results were analysed via an in-house MATLAB program based on the deconvolution process described elsewhere [27], [38]. The properties of the four working liquids are given in Table A1.

## 3 Results and discussion

### 3.1 High-frequency regime

**Fig. 1:**
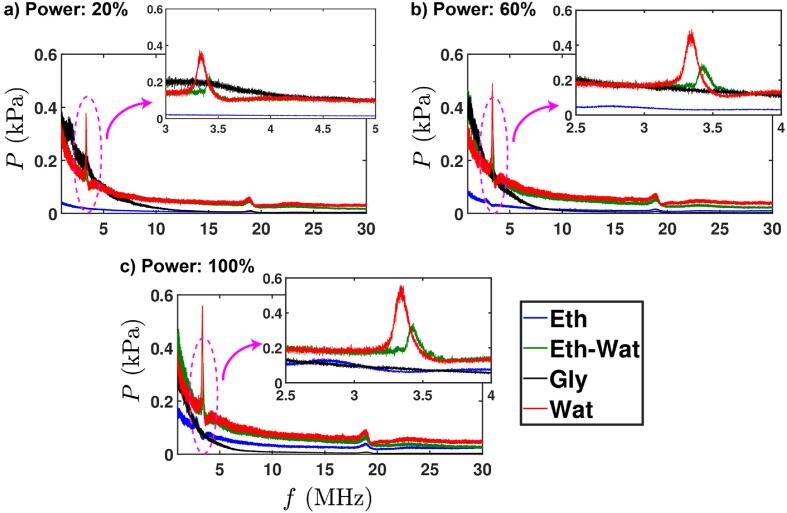
Pressure vs frequency for all tested liquids for a specific sensor position (*x* = −3, *y* = 1 mm) and different input powers: a) 20 %, b) 60 % and c) 100 %. Each inset magnifies the plot near the prominent peak frequency. The pressure was averaged over 60 waveforms. Note the same Y-axis scales in all main and inset plots.

The main objective of the current work was to study the prevalence of the strong pressure peak signal (already observed in water [27]) in the MHz range in the various liquids. This would shed light on the behaviour of shock waves in liquids other than water, and the effect the physical properties have on their propagation and their effect on overall pressure levels and cavitation zone build up. To this end, in Fig. 1 we compare the pressure spectra in the calibrated frequency range (1–30 MHz) of our sensors (associated with cavitation emissions and traveling shock waves) of the four liquids for a given sensor position (*x* = −3, *y* = 1 mm) and for input powers of 20 %, 60 % and 100 %. For clarity and consistency in this work, this position was chosen for representation and analysis for all liquids, as it is at the edges of the sonotrode which was previously shown [27] to receive undisturbed shock emissions from the collapsing bubbles. For each plot, the pressure was averaged over the 60 signals (each 2 ms of collection time) and inset plots show the magnified portion in the desired peak range. Distinct peaks were observed for water and ethanol–water solution for all input powers. In contrast, a weak and hardly distinguishable peak was recorded for ethanol and no peak at all for glycerol. In water, the peak frequency was at 3.32 MHz, 3.35 MHz and 3.34 MHz for 20 %, 60 % and 100 % input power respectively, showing that input power is not really affecting this frequency. The corresponding values for ethanol–water solution were 3.41 MHz, 3.43 MHz, and 3.43 MHz. For this solution, a clear shift of the dominant peak towards higher frequencies is captured at all input powers indicating a potential effect from the liquid environment. In most of the studied cases glycerol showed no peak (possibly due to the attenuation of the shock wave).

Previous investigations [39], [40], [41] have also focused on the detecting the shock waves emanating from the cloud of bubbles for understanding their spectral features across the frequency spectrum using different hydrophones. Though depending on the experimental conditions and the hydrophone’s features such as spatial sensitivity, bandwidth etc. the acoustic emissions could comprise either some or all of the frequency components. Sub-harmonic, ultra-harmonic and broadband (white) noise spectral peaks correspond to emissions from chaotically, asymmetrically oscillating bubbles, and shock waves emanating from transiently collapsing bubbles. On the other hand, the stably oscillating bubbles may not produce broadband noise rather distinct peaks as shown in [42], [43], [44]. However, the hydrophones utilised for capturing the acoustic emissions in aforementioned studies may not have been adequately designed for detecting shock wave emissions owing to technological limitations. For example in [41], the experiments were performed using an ultrasonic source operating at 1.075 MHz with a standard non-calibrated hydrophone (thus missing information on sensitivity variation at MHz frequencies) while an analogue spectrum analyser was used to record the signal. Analogous to previous studies, this frequency peak results not from stable oscillations or single implosions but from the cumulative collapse of bubble and bubble clouds, and the energy of shock wave emissions is distributed across the whole spectrum, i.e., from kHz to MHz range as explained in [27]. Specifically, it was shown that only a small fraction of this energy is responsible for raising this peak with the majority distributed across the full broadband frequency range. However, the presence of this peak can be an important feature for in-situ monitoring of sono-processes such as the synthesis of 2D nanomaterials as shown in [9]. Additionally, the FOH sensors used in our study have almost linear sensitivity in the frequency range of interests, 1–5 MHz (see Fig. S1 in [27]), which means there is no structural resonance of hydrophone itself. An interesting observation is that the amplitude of the shock waves is also affected by the liquid environment and to a lesser extent, the input power. It is evident from Fig. 1 that water showed the strongest peak intensity followed by ethanol–water solution with a ∼40 % decrease in the pressure peak amplitude and finally the ethanol with almost 90 % decay in the pressure peak amplitude, irrespective of the vibrational amplitude of the sonotrode.

In order to observe the shock propagation across the entire treatment domain for different input powers, a full map indicating the presence of the pressure peak in the four tested liquids for all sensor positions is shown in Fig. A2 (in the Appendix). Table 1 shows the percentage of the positions in our experimental map (as per Fig. A2) that include the distinct peak in the pressure spectrum. For water, the peak was observed for 100 % of all positions and all input powers, confirming the previous study [27]. For ethanol, we observed no peak in any position at 20 % input power, but this was increased to 62 % and 100 % of all positions with increasing the input power. For the ethanol–water solution, we observed the peak for ∼ 85 % of the sensor positions for all input powers. However, for glycerol, we observed the pressure peak only for 10 % (at 20 % power) and 4 % (at 60 % power) with no peak at 100 % of the positions. Due to the little probability of detecting the peak, we avoid any direct analysis of the frequency peak for this liquid, that could lead to misleading conclusions, since due to the viscous nature of the liquid, there is rapid attenuation of shock waves through the energy dissipation in this medium.

**Table 1:** Percentage of the positions with distinct pressure peak for 4 liquids and each input power.

	Working Liquid			
Input Power	Ethanol	Eth-Wat	Water	Glycerol
Input Power: 20 %	0	84 %	100 %	10 %
Input Power: 60 %	62 %	85 %	100 %	4 %
Input Power: 100 %	100 %	87 %	100 %	0

**Fig. 2:**
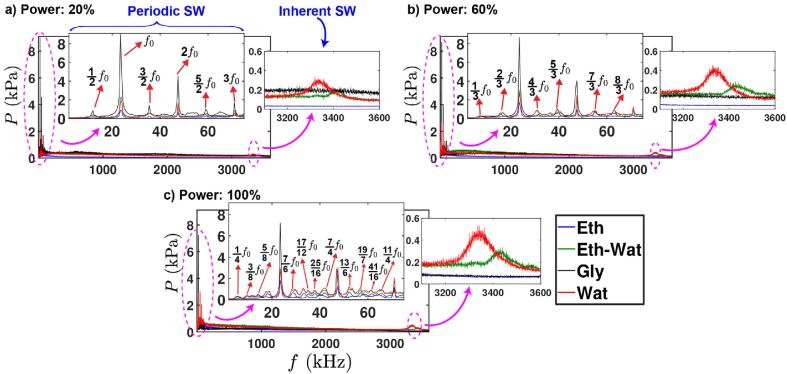
Frequency response of acoustic emissions from kHz to MHz range for four working liquids at three input powers: a) 20 %, b) 60 % and 100 %. The top insets show the zoomed-in view of the low-frequency peaks (periodic freature), while the right insets show that of the high-frequency zone (inherent feature). Note that the sensitivity at 1 MHz was used for measuring the pressure amplitudes for *f* < 1 MHz. The zoomed-in view of the low-frequency peaks in log-scale is shown in Fig. A6 in the Appendix.

**Fig. 3:**
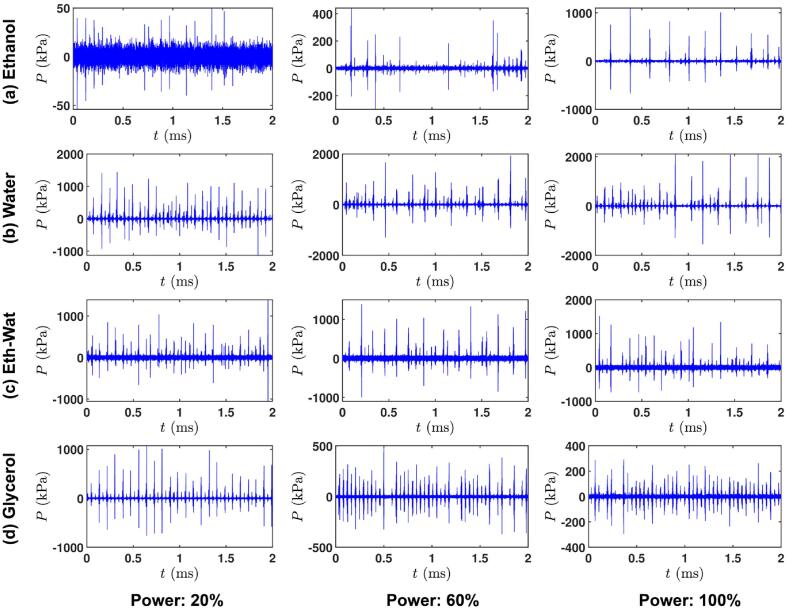
Typical pressure vs time plots for the four working liquids at three input powers: 20% (left column), 60% (middle column) and 100% (right column). Peaks are associated with the intensity of shock waves (with their inherent frequency discussed in Fig. 1). It should be noted that plots are corresponding to cases where the distinct peak at MHz frequency was also present in accordance with Table 1 and Fig. A2.

This peculiar behaviour for glycerol could likely be due to the rapid attenuation of the energy that the shock waves are carrying out. Most of the energy is dissipated to the bulk liquid causing broadening of the propagating shock wave front and the attenuation of the shock wave intensity is rapid due to the viscous nature of the liquid that also diminishes the establishment of fully developed cavitation zone [44]. This may be further enhanced by the bubbly envelope formed around the cavitation zone and close to the sonotrode tip where the emitted shock fronts from the bubble implosions are cushioned and trapped [34], [44]. Thus, even if shocks are observed as sharp periodic peaks (Fig. 3d), they are of low intensity, and possibly reaching the tip of the sensor at ∼3 MHz frequency with a diminished energy and therefore they won’t be strong enough to raise the inherent peak (Fig. 2). However, they contribute to the broadband noise and low frequencies as per Table 2. In case of liquids with the smallest surface tension such as ethanol, the cavitation bubbles may survive for a longer period owing to their large vapour pressure [44]. Thus, apart from the formation of the bubbly clouds (due to vapor cavitation) near the source, these bubbles do not tend to collapse at low (20 %) input power (but move around continuously) and disrupt the propagation of shock waves by blocking and absorbing their energy as shown in [33]. Also the speed of sound in the medium surrounding any bubble might be diminished by the presence of neighbouring bubbles or the bubbly envelopes (i.e., the fluid appears to be compressible). Based on the statistics presented in Fig. A2 and Table 1, it could therefore be interpreted that the cavitation bubble collapses are less powerful in ethanol and, in behaviour quite unlike those in water and ethanol–water solution. Whereas in case of glycerol, bubble collapses can lead to intense pressure peaks close to the horn tip [34], but they exhibit a weak cavitation regime due to large viscous energy dissipation and restricted bubble nucleation [44].

**Table 2:** Measured frequecny for major and minor peaks along with their standard deviation in each liquid and input power in Fig. 3.

Power	20 %		60 %		100 %	
Frequency (kHz)	***f*_maj_**	***f*_min_**	***f*_maj_**	***f*_min_**	***f*_maj_**	***f*_min_**
Ethanol	11.7 ± 0.2	25.5 ± 3	9.7 ± 2.8	24.2 ± 3.9	5.7 ± 1.1	23.4 ± 1.8
Eth-Wat	11.8 ± 0.7	23.9 ± 2.1	7.9 ± 0.4	23.3 ± 1.6	11.3 ± 2	23.7 ± 3.4
Water	11.9 ± 0.6	23.4 ± 3	7.2 ± 1.2	21.9 ± 2.2	9.5 ± 2.5	25.3 ± 4.1
Glycerol	11.8 ± 0.7	23.8 ± 2.2	24.2 ± 1.9	48.1 ± 6.3	23.8 ± 2.3	52.4 ± 6.6

### 3.2 Low-frequency regime

In addition to the prominent peak at high frequency (MHz), the shock waves are also associated with the strong sub-harmonic signals emitted from acoustically driven periodic bubble collapses [13], [18] exhibiting their dual origin in the spectrum. Thus, we now turn our interest to the low frequency (kHz) spectrum where strong sub- and ultra-harmonic peaks have been observed. For determining the role of periodic shock waves induced by the bubble cloud collapses beneath the sonotrode tip, a study by Song et al. [15] suggested that the energy of shock wave emissions contributes to the frequency (*nf_sw_*) spectrum at all values of *n*. Fig. 2 shows the frequency response of cavitation emissions for three input powers for the same sensor position as in case of Fig. 1 (*x* = -3, *y* = 1 mm). Each plot includes two insets magnifying the spectral peaks observed at both low (kHz, top inset) and high (MHz, right inset) frequencies for each working liquid. It should be noted here that the pressure values in the kHz range of Fig. 2 were obtained using the calibrated sensitivity at 1 MHz, hence the plots were only qualitatively assessed and compared.

At 20 % input power (Fig. 2a), the acoustic spectrum exhibited distinct peaks at sub-harmonics, fundamental, ultra-harmonics and harmonics as *nf*_0_/2 for *n* = 1, 2, 3, …*n*. As it can be seen, the shock wave emissions contributed to the spectral peaks occurring at every half (1/2 *f*_0_) interval of driving frequency. This represents the *periodic feature* of the shock waves [15], [18]. The driving frequency and the harmonics (*nf*_0_) appeared to have high pressure amplitude due to scattering of the primary wave field by the propagating shock waves as previously observed by Yusuf et al. [18]. On the other hand, the observed spectral peak at high frequency (Fig. 2, right inset) represents the *inherent feature* of the shock waves as previously discussed [27]. Interestingly, among all liquids, and in contrast to the high frequency spectrum analysis, glycerol showed very distinct *periodic* peaks with much higher amplitude (∼4 times) than others.

**Fig. 4:**
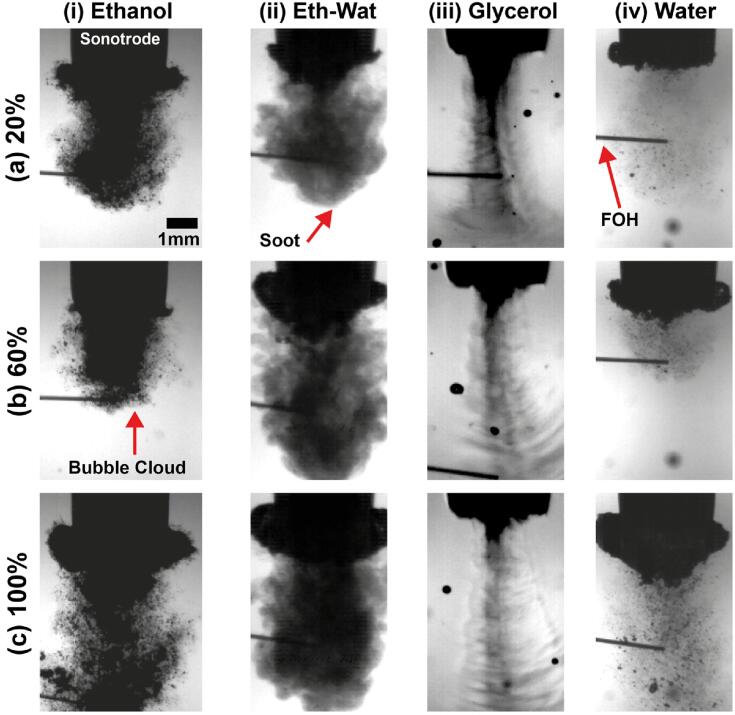
Sample snapshots from the high-speed recordings of the largest cavitation clouds of four liquids at three input powers: a) 20%, b) 60% and 100%.

We suspect that this observed peak periodicity for glycerol is attributable to the formation of the standing waves (see imprints of waves on the background of Fig. 4c as well as the corresponding Viddeos 2 and 3) plus the over-imposed shock waves (see Fig. 3) that propagate at this location (*x* = -3, *y* = 1 mm) owing to lower cavitation shielding offered by the smaller cavitation zone with attenuated bubble collapses [44] as a result of increased viscosity and lower vapour pressure [34]. Water and ethanol–water solution exhibited almost similar behaviour, while ethanol showed weak spectral response at all broadband frequencies. Supplementary Viddeos 1–4 show a representative sequence of shock wave propagation for water, ethanol and glycerol at 20 % input power. It is clear that water generates a significant amount of shock waves that propagate far within the liquid medium. On the contrary, glycerol exhibits some amount of periodic shock waves that do not seem to propagate far, as they rapidly lose their energy in this highly viscous liquid (note that they travel faster in glycerol than other liquids, therefore the camera may not be able to capture all the “faint” due to rapid energy loss of shock waves). On the other hand, ethanol has virtually negligible generation of shock waves. Hence, the MHz peak for these 2 liquids is suppressed, as seen in the video recordings and reported in Table 1 and Fig. A2. In addition, it is interesting to see that no bubbly structures are formed in glycerol as expected (and thus less shielding effect close to the tip), while numerous scattered bubbly patterns are formed in the bulk ethanol environment. Note that in the case of the low frequency regime (periodic feature), no shifting of the peak was observed, unlike the shift in ethanol–water solution for the high frequency peak (Fig. 1). This is possibly associated with the speed of sound in the medium (that most probably governs the frequency shift) rather than frequency of the source. At low driving amplitudes, cavitation activity is much more controlled as the periodic collapses are regulated by the fundamental frequency of the ultrasound i.e., 24 kHz.

At 60 % input power (Fig. 2b), we observed a similar behaviour, however the sub-harmonic, fundamental, ultra-harmonics and harmonics are observed as *nf*_0_/3, i.e., bubble collapses occurring at every 1/3 interval of the driving frequency (see also Table 2). Finally, at 100 % input power (Fig. 2c), we observed a greater number of sub-harmonic and ultra-harmonic peaks for all liquids (compared to 20 % and 60 %), except glycerol. This was accompanied by distortion in the periodicity of the cluster collapses (and thus of the shock emissions) leading to irregular spectral peaks. The distortion may be a result of increased occurrence of non-collapsing deflations [18]. It has been previously reported that at higher input powers, the shock wave periodicity in water becomes less dominant at both transitional (primary bubble cloud oscillations at sub-harmonic frequencies) and non-transitional input powers caused by the variance in amplitude of the multi-fronted shock waves raising the noise floor in the spectrum [18], [45]. This is also evident from Fig. 3b where shock peaks in water especially at 100 % are mixed with sparse irregular high and low peaks. However, the major frequency peaks periodicity it is still associated with the rise of the sub-harmonics (see also Table 2). At this high input power, glycerol exhibited only the fundamental peak and harmonics with lower intensities compared to the lower input power. The reason for the suppression of sub-harmonics and ultra-harmonics for glycerol at 60 % and 100 % power was possibly the weakening of shock waves as they travel through the bubbly envelopes [44], though more in numbers as Fig. 3d and Supplementary Viddeo 3 (glycerol at 60 % power), and that the regimes at high input power are dictated by fundamental waves. Thus, the sub-harmonic peaks in mediums with low shielding effects e.g., low input power, promote regular bubble collapses at similar intervals irrespective of the liquid environment showing similar sub-harmonic frequency peak pattern. Hence, bubble collapses in those regimes are regulated by the fundamental frequency and acoustic intensity from the source.

The low frequency components in glycerol represent the major contribution from the direct field emissions and associated harmonics. However the raise of the sub-harmonic component such as in the case of 20 % as well as any ultra-harmonics captured at higher input powers indicates contribution from periodic shock waves as explained in [15]. In addition, each of the harmonic peaks also represents the contribution from the non-inertial (stably oscillating) cavitating bubbles and is associated with a specific mode shape that includes the characteristics of standing wave field. Rapid damping in the acoustic wave energy due to high viscosity of the medium leads to increase in the oscillation period of stable cavitation bubbles superimposed on the driving frequency and further contributing to harmonics and ultra-harmonics spectra features.

### 3.3 Pressure distribution

The rising of periodic shock wave frequency peaks in the acoustic spectrum derives from the real-time distribution of the shock pressure peaks observed in the pressure–time domain. We show representatives of these *P-t* plots for a given signal for our four working liquids and each input power in Fig. 3. This figure shows distinct high (major) and low (minor) pressure amplitude peaks spaced across the waveform of 2 ms duration for each case. Each liquid exhibited alternating major and minor peaks, where the major peaks are mainly associated with the primary periodic cluster collapses at different frequency intervals i.e. *f*_0_/2, *f*_0_/3 etc. (where *f*_0_ is the driving frequency) attached to horn tip emitting multi-fronted shock waves at sub-harmonic frequencies apart from glycerol at 60 and 100 % (major peaks associated with frequencies of the incident wave) as shown from Table 2. Whereas, the minor peaks are mainly related with emissions at the driving frequency (apart from glycerol that relate to harmonics) with further contributions from sub-cluster and satellite clusters collapses of cavitating cloud away from the tip emitting weak shock fronts raising ultra-harmonics and broadband frequencies as reported before [18]. Any extended duration between the shock pressure peaks could be a result of non-collapsing deflations or shielding. Increase in the input power also resulted in the increase in the number of pressure and frequency peaks both in time and frequency domain, respectively. Table 2 shows the measured frequency of shock pressure peaks arising from the major and minor peaks observed in Fig. 3.

**Fig. 5:**
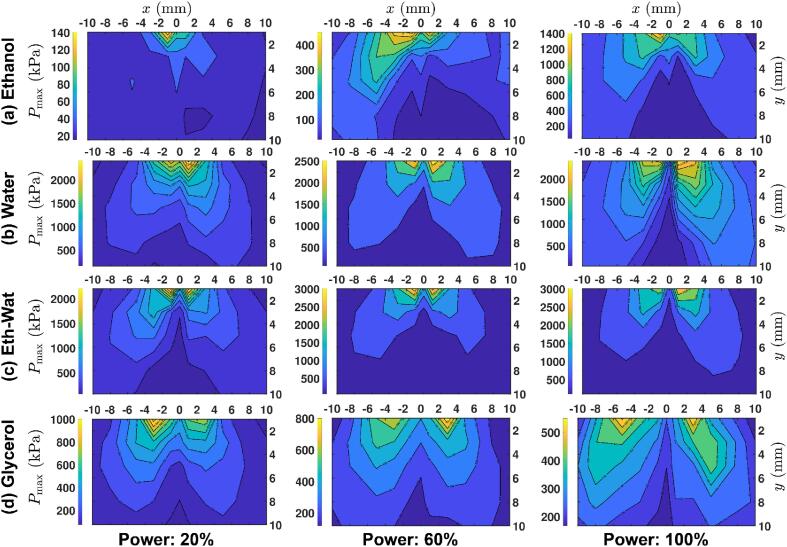
Contour plots of the maximum pressure (*P*_max_) for a) ethanol, b) water, c) ethanol–water solution and d) glycerol, each for three input powers: 20% (left column), 60% (middle column) and 100% (right column). The sonotrode is axisymmetrically placed at (0,0).

At all input powers, the major and minor peaks appeared to be equally spaced in time across the whole waveforms with major peaks contributing to sub-harmonic frequencies and minor peaks to fundamental frequency (see Table 2). It is also interesting to note that in every approximately 2–3 acoustic cycles a large pressure peak is observed in water and ethanol–water solution while in ethanol, the peaks are more erratic and in glycerol more consistent though weak and correspond to the incident frequency. In specific, and according to Fig. 3 for all liquids, at 20 % input power the peak is seen every 2 cycles. For the water, ethanol, and ethanol–water liquids with increase of power input to 60 % and 100 % collapses repeated every 2–4 cycles (though with slightly irregular periodic pattern for 100 %) implying the effect of shielding as more bubbles are formed beneath the tip with the increase of power (see also Fig. 5) while non-collapsing deflations may occur as discussed earlier. For glycerol with increase in input power to 60 % and 100 %, the occurrence of major peaks was substantially reduced and appeared to be replaced with equidistant minor peaks corresponding to the fundamental and ultra-harmonic frequencies. It is evident that the higher the input power, the larger the number of pressure peaks that correspond to the fundamental frequency. This implies that regular collapses at every acoustic cycle (of 41.6 µs) are occurring. This seems to be true according to Fig. 5 as shielding effect has not yet fully developed in the glycerol at those power levels and thus multiple shock waves of lower intensity, due to bubbly envelopes, are travelling in the bulk medium. Furthermore, the small frequencies observed (e.g. at 100 %) for ethanol are likely to be related with the large long-lived ethanol vigorously pulsating bubbles (as previously observed in [44]) as well as the disruption of the shock waves from the cloud of bubbles and individual bubbles in the liquid bulk that block the propagation of shock waves and thus increase the time needed for a shock wave to reach the sensor tip (note the big time gaps between major peaks for ethanol in both 60 % and 100 %). But at 20 % power, due to the weak cavitation zone, not many bubbles, that can fluctuate vigorously, are formed and therefore, no small frequency (but merely a regular *f*_0_/2) as well as high frequency (MHz) peak (due to the very weak intensity peaks, Fig. 1a) is observed.

We can also infer from Table 2 that the major frequency peak seems to be almost half or a third of the minor one for most cases (except ethanol at 100 % power that is *f*_0_/4). We might hypothesize that the periodicity of the shock waves that raises the major peak (sub-harmonically or close to the fundamental frequency as in the case of Glycerol at 60 % and 100 % power) may be the reason via its corresponding harmonic that raises the minor peak as well. Also, it is clear from Table 2 that for all input powers (but especially at 20 %), most of the peaks are associated with the rise of sub-harmonics (apart from glycerol at 60 % and 100 %), implying a clear contribution from the shock waves in that frequency range. Therefore, at low powers where shielding is less, the shock waves are resolved better with strong sub-harmonic signatures. This can also be attributed by the standard deviation as per Table 2 where it stays low for low input powers and increases for higher input powers implying the presence of a more variable (chaotic) cavitation regime (as also depicted in Fig. 2c). For ethanol and glycerol, the shock waves are weak and for glycerol, they decay even faster, while for water and ethanol–water solution, they remain strong and raise the MHz peak.

The appearance of sub-harmonics and ultra-harmonics is also influenced by the oscillation frequency and size of the bubble clouds attached to the sonotrode tip. Fig. 4 shows representative snapshots of the cavitation bubble clouds for the four working liquids at three different powers (see corresponding videos 5–8 for clarity). Note that the bubble clouds attached to the tip usually are formed within roughly 5–6 ms after the sonotrode was activated [28]. As can be seen, typically for the majority of liquids, the bubble cloud just beneath the sonotrode tip was enlarged with increasing power, except for glycerol where the cloud shrank, forming viscous streams with the formation of standing waves. Note that when the incident acoustic waves emitted from the ultrasonic transducer undergo reflection at the walls of the vessel and/or the liquid surface, standing wave patterns can form. They can form in any liquid medium when the distance between the ultrasonic emitter and the reflecting surfaces/walls is equal to multiples of half of the wavelengths of the travelling wave, which can be altered with the liquid level in the vessel. With the formation of standing waves, there is no directional transmission of the acoustic energy within the medium and the energy transfer is present only at nodes and antinodes.

For ethanol (Fig. 4 (i)), more satellite bubble clusters were distributed away from the horn. On the other hand, the size of satellite bubbles away from the tip were different for various liquids, that is, the bubbles in ethanol were larger than those in water and ethanol–water solution (see videos 5, 6 and 8).

In the case of ethanol (Viddeo 5), although the sub-harmonic and ultra-harmonic frequency peaks at 100 % input power are clearer and have higher amplitude than those at 20 % and 60 % (possibly induced from the oscillation frequency of the main bubble cluster attached to the horn tip), the overall bubble collapses are less due to the large volume fraction of long-lived bubbles that also cushions the propagating shocks waves. Another important factor in the cavitating bubble dynamics and acoustic wave propagation could be phase change. The presence of cavitation bubbles lowers the propagation speed of an acoustic wave that can significantly affect overall pressure field within the liquid due to compressibility, where the shock fronts emitted from the bubble collapse travel at velocities close to the speed of sound. As the ultrasonic waves propagate inside a bubbly liquid, the bubbles tend to oscillate in response. Simultaneously there is attenuation of acoustic energy and reduction in wave speed owing to bubble pulsation, which has been confirmed using theoretical modelling based on the linear wave propagation and has been seen to agree well with experimental observations [46], [47], [48], [49]. However, it has been reported that the attenuation is much stronger in the case of non-linear wave propagation than that of a linear wave The effective wave speed and attenuation can vary with the amount of vapour, gas or their combined mixture present in the liquid. Variation in the wave velocity as a result of phase change becomes very complex as the void fraction in liquid increases. Owing to the high volatility of ethanol, the void fraction increases with the increase in ultrasound input power, leading to the generation of more long-lived bubbles pulsating in a stable linear or non-linear fashion, which alters the shock wave propagation drastically as seen in [50]. Probably, that is why the shock pressure peaks are much more erratic as seen in Fig. 3 with minor peaks contributing mainly to the driving frequency component (see Table 2).

Water and ethanol–water solution exhibited similar cavitation behaviour in the primary cluster, increasing with input power. In the ethanol–water solution, an interesting mist-like shape dominated the cavitation zone and became denser with increasing power (see ethanol–water in Fig. 4 and Viddeo 6). The intensity of the frequency peaks however was found to be smaller at large input powers implying a less aggressive cavitation environment with many tiny bubbles forming a mist-like pattern in contrast to water, where the intensity increased with increasing power at both low and high frequencies.

Qualitative results from high-speed video recordings in Fig. 4 revealed that based on the properties, each liquid behaves differently with water, ethanol–water and ethanol raising sub-harmonic peaks (related to shock waves). The peaks at water and ethanol–water solution show a similar pattern in their cavitation behaviour (Fig. 3), however the determining factor is the mist-like feature of the solution that maybe beneficial for processes related to exfoliation of nanomaterials (while maintaining their quality and integrity) where a gentle exfoliation from tiny bubbles is desired [28].

The cavitation-induced shock wave propagation can significantly alter the maximum acoustic pressure (or shock pressure) field in the surrounding medium. This has been explained in Fig. 5, where we show the shock pressure distribution (for the calibrated range of 1–30 MHz) across the cavitation zone via contour plots of the maximum pressure vs sensor positions for all four liquids and three input powers (the corresponding contour plot for root-mean-square (RMS) pressure is given in Fig. A3 in the Appendix). For every liquid at each input power, the maximum shock pressure was found to be substantially higher in regions close to the tip of ultrasonic source, where the bubble cloud size was largest (see Fig. 4), indicative of a strong non-linear inertial cavitation regime. The shock pressure was however observed to maximise closer to the side edges of the sonotrode rather than just below the centre of the horn tip where the cavitation activity is more rigorous. This has been previously attributed to the cavitation shielding effect [27], [51]. Cavitation (acoustic) shielding [44], [52], [53] also causes the pressure field to attenuate drastically within the cavitation zone as we move away from the sonotrode. Visually, shielding can be identified as the lowest pressure region below the centre of the horn surface that becomes prominent as the vibration amplitude of the sonotrode is increased (see Fig. 5a, b and c).

To physically quantify the shielding effect and compare its relative response for each liquid, we introduced a term called *shielding factor*, defined as the percentage of the normalised ratio of maximum shock pressure generated in the vicinity of the source to that of shock pressure in the bulk liquid away from the source (∼10 mm below the horn) with the relevant data presented in Table A2. Ideally, a large shielding factor would mean that the cavitation activity is larger with powerful bubble collapses near the ultrasonic horn, thereby increasing the chances of more shock front cushioning/absorption and thus large pressure attenuation in the bulk liquid. Table 3 shows the shielding factor calculated for different liquids at 20 %, 60 % and 100 % input power.

**Table 3:** Shielding factor of 4 liquids at each input power derived from the pressure data of Table A2.

Power	20 %	60 %	100 %
	**Shielding factor (%)**		
Ethanol	14.5	41	81.8
Eth-Wat	70	100	56.5
Water	21.2	88.6	96.7
Glycerol	25.5	11.2	6.6

Interestingly, shielding becomes more dominant with the increase of input power in all the liquids as previously reported [27], [44], [51], except glycerol which shows the opposite trend. It can also be seen from Table 3 that among all liquids, shielding factor is maximum for ethanol–water solution at 60 % input power while is lowest for glycerol at 100 % where the shock waves are rather weak and dictated by the strong fundamental waves. At 20 % input power, ethanol (Fig. 5a) showed the smallest shielding factor. This could be related to the weak acoustic pressure field generated because of the low energy implosions of the cavitating bubbles due to their low surface tension and low viscosity (see Viddeo 1). Ethanol’s shielding factor increased to 41 % at 60 % power and ∼82 % at 100 % power. Moreover, in ethanol, the contour plot is also very asymmetric at low input powers (20 % and 60 %) and is likely to be related to the high instability of bubble structures generating dispersed and satellite clusters that vigorously oscillate themselves, rather than complicated bubbly structures with multiple collapsing events (see Viddeos 1 and 5). Ethanol-water solution (Fig. 5c) in contrast, showed the largest shielding factor at 20 %, even more than in water (Fig. 5b). This can be attributed to the ethanol content in the solution, increasing its surface tension, as ethanol produces many tiny cavitating bubbles that create the mist-like patterns beneath the sonotrode; these likely cushion the shock wave propagation (see Fig. 4 (ii)). We have seen in many occasions the effect of surface tension in bubbles such as [54], [55], [56] where low surface tension values similar to that in ethanol generate bubbles with extended lifecycles, vigorous and irregular bubbly motion, preventing regular collapses with diminished intensity. Thus, a liquid with lower surface tension like ethanol in combination with the high vapour pressure phase is likely to have a less aggressive behaviour as this is also reflected by Fig. 3a with the sporadic shock impulses.

It should be noted that shielding factor becomes a maximum at 60 % power but then decreases by 43.5 % at 100 % power. This unexpected decrease may be a result of increased collapsing events of tiny bubbles formed within the mist-like pattern further away from the source (acoustic streaming is more powerful [36] pushing the mist away), promoting more shock propagation into the bulk, thus less absorption. This tendency of increasing cavitation intensity with input power followed by a temporary drop has been also previously seen in [18], [57]. It is clear from Fig. 5 that the maximum pressure is significantly suppressed at 1 mm below the centre of the sonotrode compared to that at the edges for all the input powers, possibly owing to the large mist-like pattern formation. In addition, the penetration depth of the low-pressure zones in the central region extended towards the horn tip with increase in power for all liquids except glycerol, with this penetration being more significant in the ethanol–water solution than other liquids (Fig. 5c). Water (Fig. 5b), among all liquids except glycerol showed the largest increase (approximately 300 %) in the shielding factor from 20 % to 60 % power. From 60 % to 100 % power, the shielding factor however only increased further by ∼9 % reaching up to 96.7 %. In glycerol (Fig. 5d), we observed the minimal shielding effect with shielding factor decreasing with power and reaching almost 6.6 % at 100 % power. This was expected as sufficiently more displacement from the horn is achieved (opening pathways for acoustic emissions to travel deeper in the bulk rather than instantly fading out due to viscous nature), being able to generate an extended cavitation zone in this highly viscous liquid, however the bubbly envelope may diminish the intensity of shock waves as discussed earlier.

In the case of water and ethanol, cavitation shielding is dictated by dominating inertial forces leading to bubble collapses. In ethanol–water, the shielding is dictated by high momentum forces indicating high susceptibility of cavitation bubbles to collapse close to the source and therefore more cushioning of the periodic shock waves from the mist-like pattern. The cavitation shielding in glycerol is dictated by high viscous forces (viscosity significantly dominating the surface tension). The latter is in agreement with the above discussion about the constriction of cavitation clouds at high input powers and the formation of sustained bubbly envelopes (see Fig. 4c (iii), video 7 and Table 2). The dominant viscous forces may also be the reason for the highly symmetric pressure profiles for glycerol. So, even though the penetration depth of the shocks inside glycerol is probably the largest among all the input powers, the absence of a clear frequency peak in the acoustic spectrum profile (Fig. 1) predicates from the energy dissipation of the propagating shocks. The broadband noise in glycerol is dominant in the frequency range of 1 – 4 MHz as per Fig. 1 that indicates the contribution of the shock waves to the noise floor rather than to the MHz peak. It is also interesting to note that increasing the input power decreased the shock pressure in glycerol close to the source, contrary to what is seen in case of other liquids.

We note here that any potential resonance do not play a major role on the strength of the generated shock impulse from bubble implosions as previously seen in [27]. At lower power settings, (i.e. 20 %), the absence of strong cavitation activity facilitates the formation of prominent fundamental peaks and corresponding harmonics as sound waves propagate unhindered within the medium. Travelling pressure waves will cause fluid motion downstream [36] followed by the formation of the standing wave patterns that observed in glycerol (Fig. 4). As the input power increases, there is a transition to the developed cavitation regime that leads to strong scattering and attenuation effects of the propagating acoustic (Fig. 2) and shock waves (Fig. 3).

Based on the pressure map in Fig. 5, we can observe that the most effective cavitation treatment zones for all liquids are the regions around the corners of the sonotrode tip where the shock pressure is more effective and seen to even break floating metallic crystals [51] in comparison to that below the sonotrode where the pressure drop is drastic.

Based on the contour maps (Fig. 5c), we can see that the shock pressure in ethanol–water solution at 100 % input power is almost 600 kPa higher than in water close to the sonotrode. However, this is in contrast to Fig. 1c, where we saw that pressure amplitude of the frequency peak is ∼40 % less. This is likely due to the formation of myriads of tiny bubbles (mist-like pattern) that pulsate vigorously raising the noise floor (broadband noise in MHz range). This can be confirmed in Fig. 1c, where the cavitation broadband noise for ethanol–water solution is above the noise produced in water in the range of 1 – 3 MHz (see the green line in Fig. 1c). Tzanakis et al. [44] have also previously observed this tendency, where long-lived pulsating cavitation bubbles in ethanol excited by a 20 kHz source contributed to the rise of spectrum in MHz frequencies. Therefore, this infused solution of ethanol–water is also expected to give rise to the MHz broadband noise via the sustained mist-like formation of tiny bubbles. Hence, the symmetrical pressure profile complemented by maximum pressures generated in ethanol–water solution at 60 % input power (Fig. 5c) can be considered as a trade-off between shielding and obtained pressure surges even though the shielding factor at 100 % power is lower than the factor at 60 %. Therefore, the combination of medium input power of ultrasound (for this specific set up with a sonotrode of 3 mm) with this infused liquid can serve as an ideal setting for applications such as nano-exfoliation where powerful pressure fields complemented by tiny bubbles are required [28], [58]. Moreover, since these pressure contour maps are reconstructed from the shock pressure peaks in pressure–time profile, which are important for controlling the production and quality of nanomaterials in sono-exfoliation [9], [28], the mist-like zone (with myriad of tiny bubbles vibrating) along with shock pressure surges could expedite such processes in an eco-friendly liquid environment. In order to compare the pressure profiles in different liquids, in Figs. A4 and A5 in the Appendix, we show the distribution of maximum and RMS pressure vs horizontal distance (of the sensor from the sonotrode) for four liquids and each of the five vertical distances. It is apparent from these plots that at locations close to the sonication source (*y* = 1 mm), the pressure magnitudes in water and ethanol–water solution (while comparable to each other) were significantly larger than in the other two liquids. As we move away from the ultrasonic source, we observed, as expected, a significant drop in pressure for all liquids. Also, increasing the input power, raised the pressure at most positions in general and specifically at the edges of source, except right under the sonotrode due to the shielding effect as discussed above.

## 4 Conclusions

Spatio-temporal evolution of shock wave dynamics exhibited by collapsing cavitation bubbles during ultrasound (24 kHz) exposure in 4 different liquid environments with largely different physical properties showed that:

• The high-frequency pressure peak in MHz associated with the shock wave emissions is barely observed for ethanol and glycerol, particularly at low input powers, but is consistently observed for the ethanol–water solution and water indicating an increased cavitation activity.
• We also observe a slight shift in the high frequency peak for ethanol–water solution compared to that of water due to variations in the speed of sound. Though, no shift is observed in the low frequency (kHz) regime, as prominent peaks are regulated by the frequency and amplitude of the sound source. Interestingly and for all liquids, the sub-harmonic peaks, associated with periodic shock waves, were prominent at low power inputs where the shielding effect was weak.
• The studied liquids show distinctly different cavitation zone characteristics in terms of both shock pressure amplitudes generated by the periodic bubble cloud collapses and shielding factor caused by the absorption/cushioning of propagating shock fronts that affect the development of the acoustic field. In particular, high-speed images of ethanol–water solution showed an interesting pattern of a mist-like cavitation zone formation accompanied by the highest pressure profiles. This environment can be used for processes that require effective treatment such as the exfoliation of nano-materials.
• Shock pressure analysis showed that the acoustic field near the tip is much more powerful in ethanol–water solution with increase in pressure surges by 400–600 kPa than water at high input powers in spite of high shielding factor caused by the mist-like pattern formation. Acoustic shielding is most significant for the ethanol–water solution at 60 % power and prevents pressure distribution deep in the bulk liquid whereas glycerol shows almost no shielding effect presumably due to its high viscosity.
• In liquids with low viscosity (as in water, ethanol and ethanol–water solution), the surface tension and inertial forces would dictate the cavitation dynamics thereby raising the frequency peak effectively. However, in highly viscous liquids such as glycerol, the viscous forces would be dominating over the surface tension and momentum forces, thus causing the frequency peak to suppress completely due to larger energy dissipation.

Results of this first time study validate our previous hypothesis made in [27] that the cavitation induced-shock waves could indeed give rise to a specific high frequency peak in the range of 3 MHz depending on the liquid environment. In the technological context, our simple experimental approach/technique provides an efficient way to characterize cavitation-induced shock wave emissions that can serve as a metrological tool for monitoring the cavitation activity in industrial systems of various scales where other alternatives such as direct observations or numerical models fail. This will be highly beneficial and of particular interest to the materials, pharmaceuticals and food processing industries as well as the physics and sonochemistry communities.

## Declaration of Competing Interest

The authors declare that they have no known competing financial interests or personal relationships that could have appeared to influence the work reported in this paper.

## Data Availability

Data will be made available on request.
