# Supplementary material for: Cavitation-induced shock wave behaviour in different liquids
Source: Ultrason Sonochem. 2023 Feb 14;94:106328. doi: 10.1016/j.ultsonch.2023.106328 (PMC9975297; doi:10.1016/j.ultsonch.2023.106328)
Supplement: Supplementary Data 1 [file mmc1.pdf]

Appendix

Cavitation-Induced Shock Wave Behaviour in Different Liquids

Mohammad Khavari, Abhinav Priyadarshi, Justin Morton, Kyriakos Porfyrakis, Koulis Pericleous, Dmitry Eskin and Iakovos Tzanakis

Table A1. Physical properties [1-3] for the four working liquids used in present study.

	Ethanol	Eth-Wat	Water	Glycerol
**Density, ρ (kg/m^3)**	785	859	1000	1260
**Surface Tension, σ (N/m)**	0.022	0.026	0.079	0.064
**Viscosity, μ (Pa.s)**	1.1×10^{-3}	2.37×10^{-3}	1×10^{-3}	0.95
**Speed of Sound, V (m/s)**	1150-1200	1540 ± 10	1480	2000-2050
**Vapour Pressure (Pa)**	5.3×10^{3}	3×10^{-3}	2.2×10^{-3}	Negligible

Table A2. Maximum and minimum values of the maximum pressure and their ratio for each liquid and input power.

**Power**	**20%**			**60%**			**100%**		
**P_{max} (kPa)**	**Max**	**Min**	**Max/Min**	**Max**	**Min**	**Max/Min**	**Max**	**Min**	**Max/Min**
**Ethanol**	153.8	20.6	**7.5**	466.2	22	**21.2**	1490.3	35.3	**42.3**
**Eth-Wat**	2290.5	68.2	**33.6**	3275.9	63.3	**51.7**	3020.5	103.5	**29.2**
**Water**	2455.5	222.9	**11**	2830	61.9	**45.8**	2417.9	48.4	**50**
**Glycerol**	1051	79.6	**13.2**	888.3	152.2	**5.8**	559.6	166.7	**3.4**

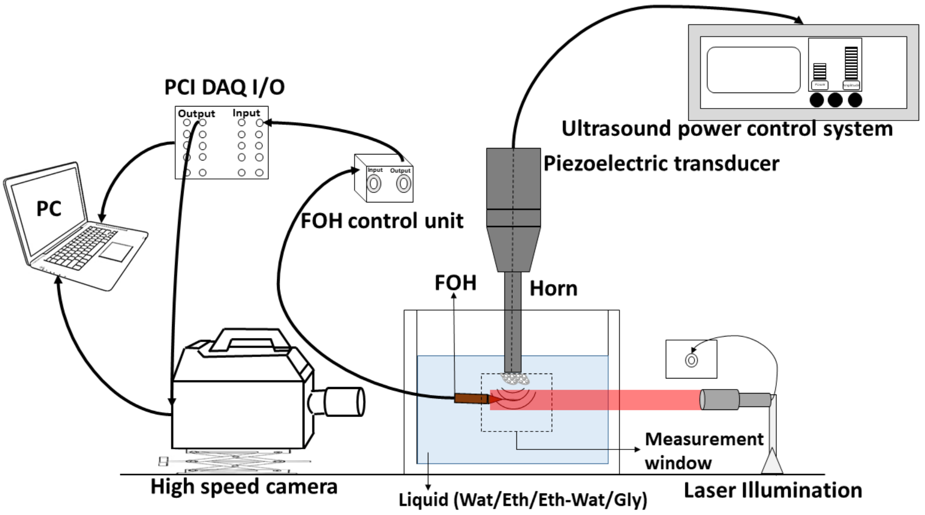

Fig. A1 Schematic representation of the experimental setup

Fig. A2 shows the complete map of the presence of the pressure peak in the four tested liquids for all sensor positions and nut powers. The map includes four different markers representing four liquids for all positions and three transducer powers. The solid markers show the presence of the peak and open markers represent no peak for the corresponding position and power.

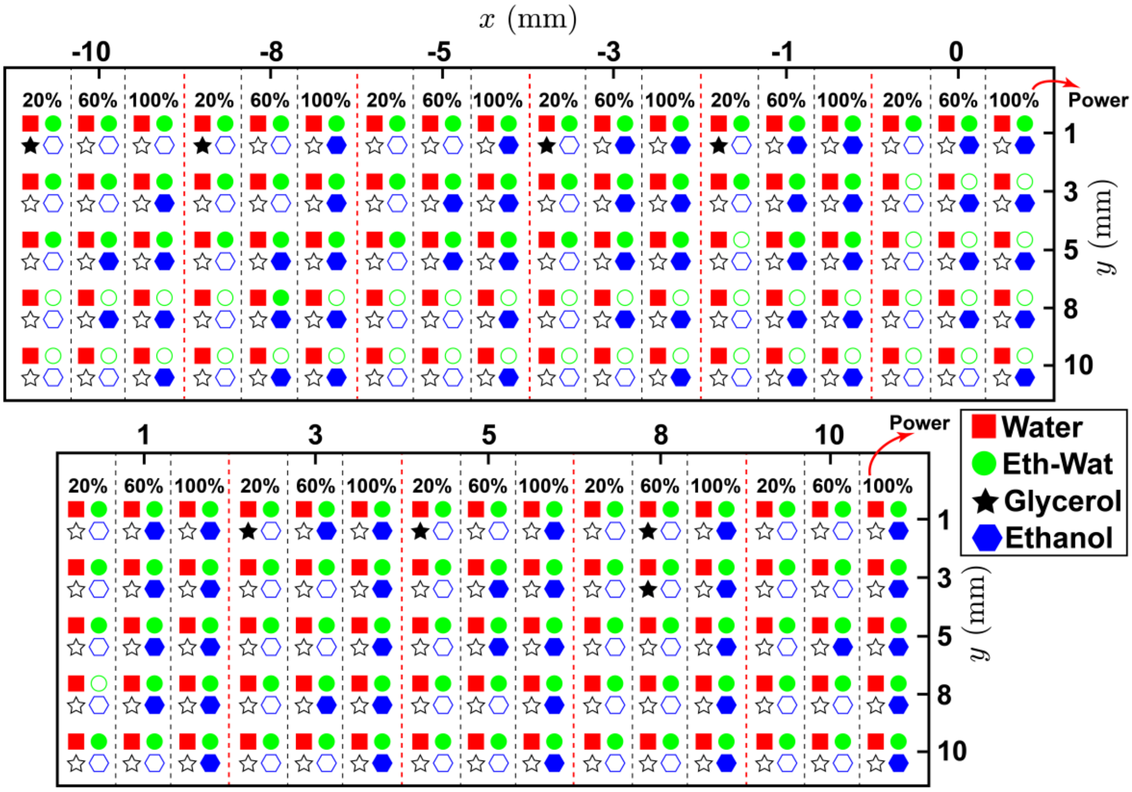

Figure A2. Complete map of the pressure peak (in the range of 2-4 MHz) for four working liquids: water (red square), ethanol-water solution (green circle), glycerol (black star) and ethanol (blue hexagon) where zero position indicates the center line of the submerged 3-mm probe while minus and plus in the x-direction represent the planes/areas left and right from the sonotode respectively. Solid markers represent the presence of the pressure peak and open markers show no peak.

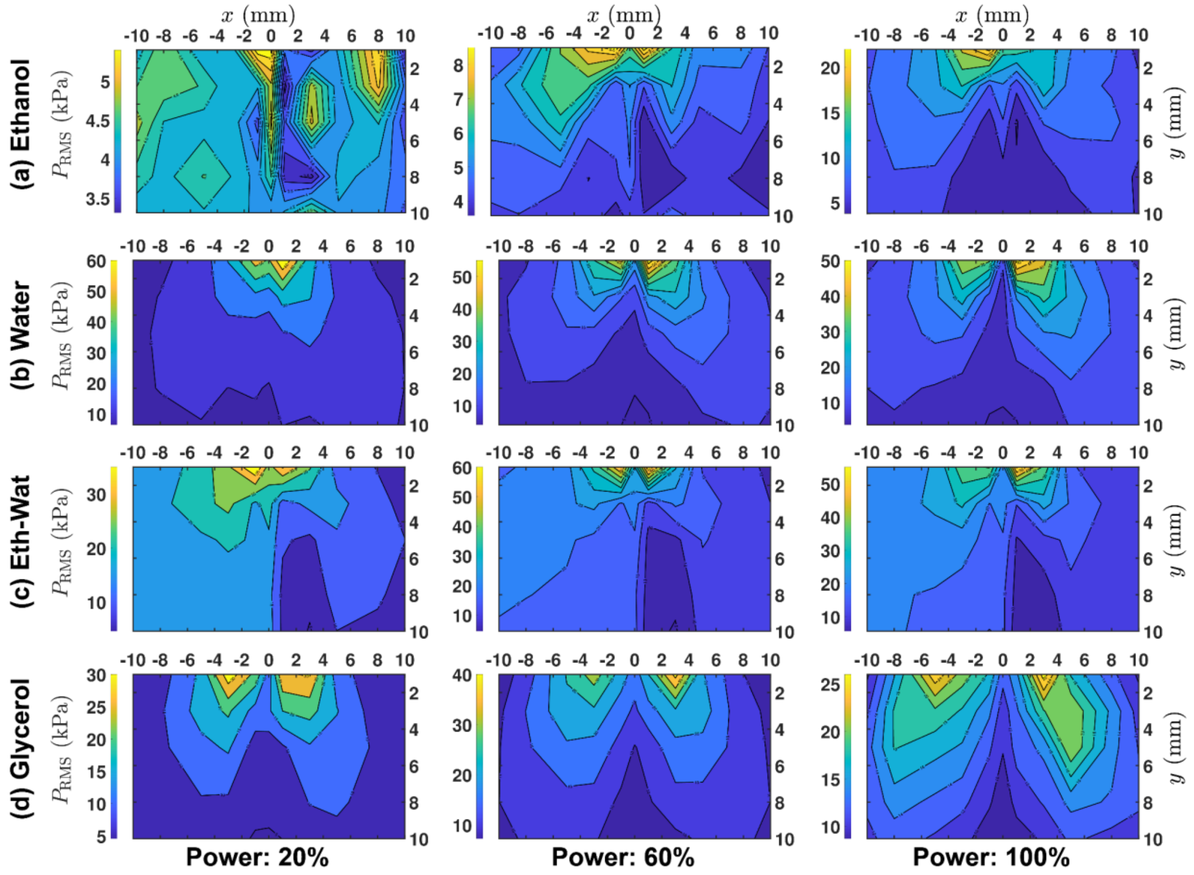

Figure A3. Contour plots of the RMS pressure (P_{RMS}) for a) ethanol, b) water, c) ethanol-water solution and d) glycerol, each for three input powers: 20% (left column), 60% (middle column) and 100% (right column). The sonotrode is placed at (0,0).

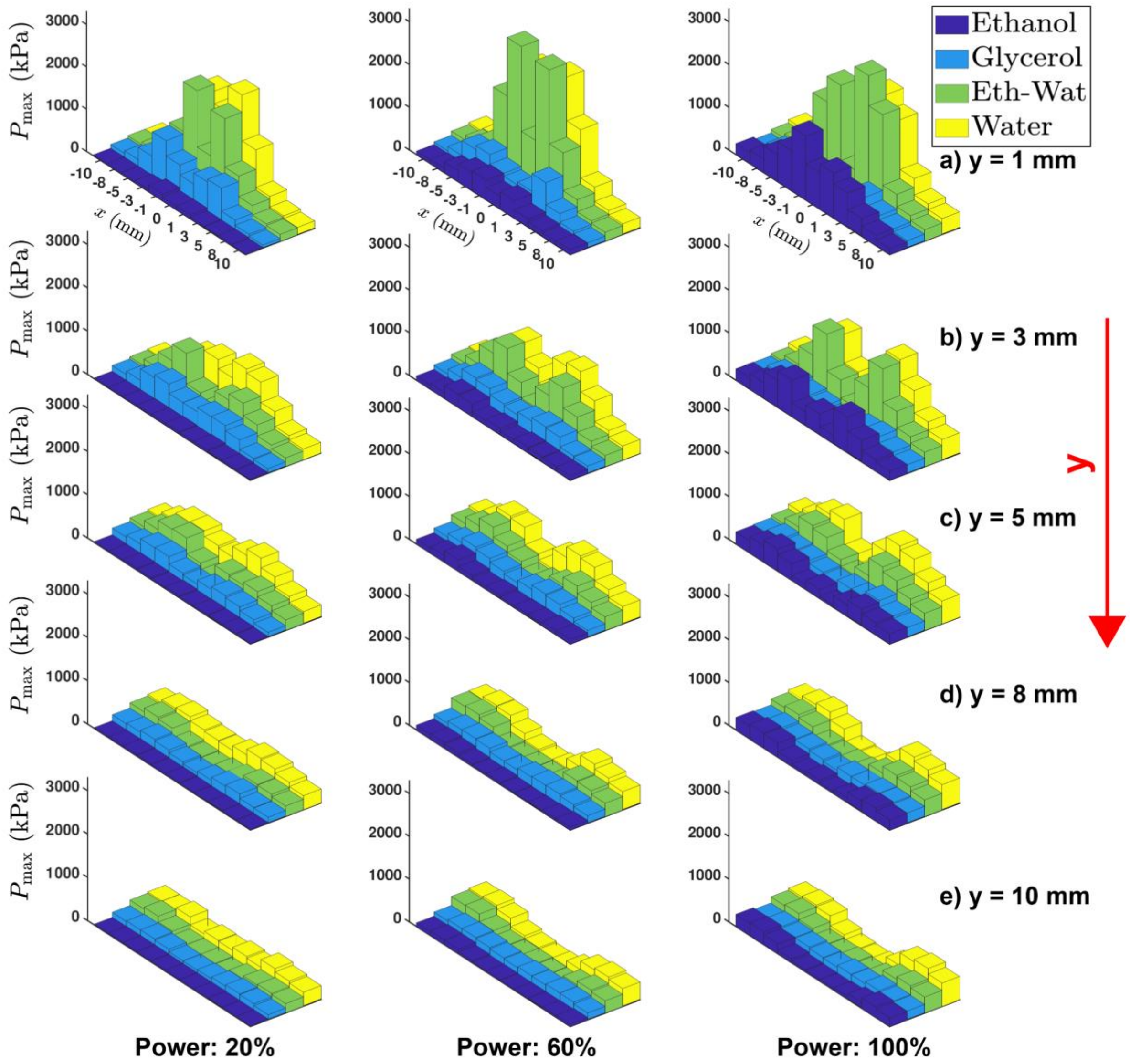

Figure A4. Comparison of the distribution of the maximum pressure ($P_{\max}$) vs. horizontal position of the four working liquids for different vertical positions (y = 1-10 mm) and three input powers: 20% (left column), 60% (middle column) and 100% (right column).

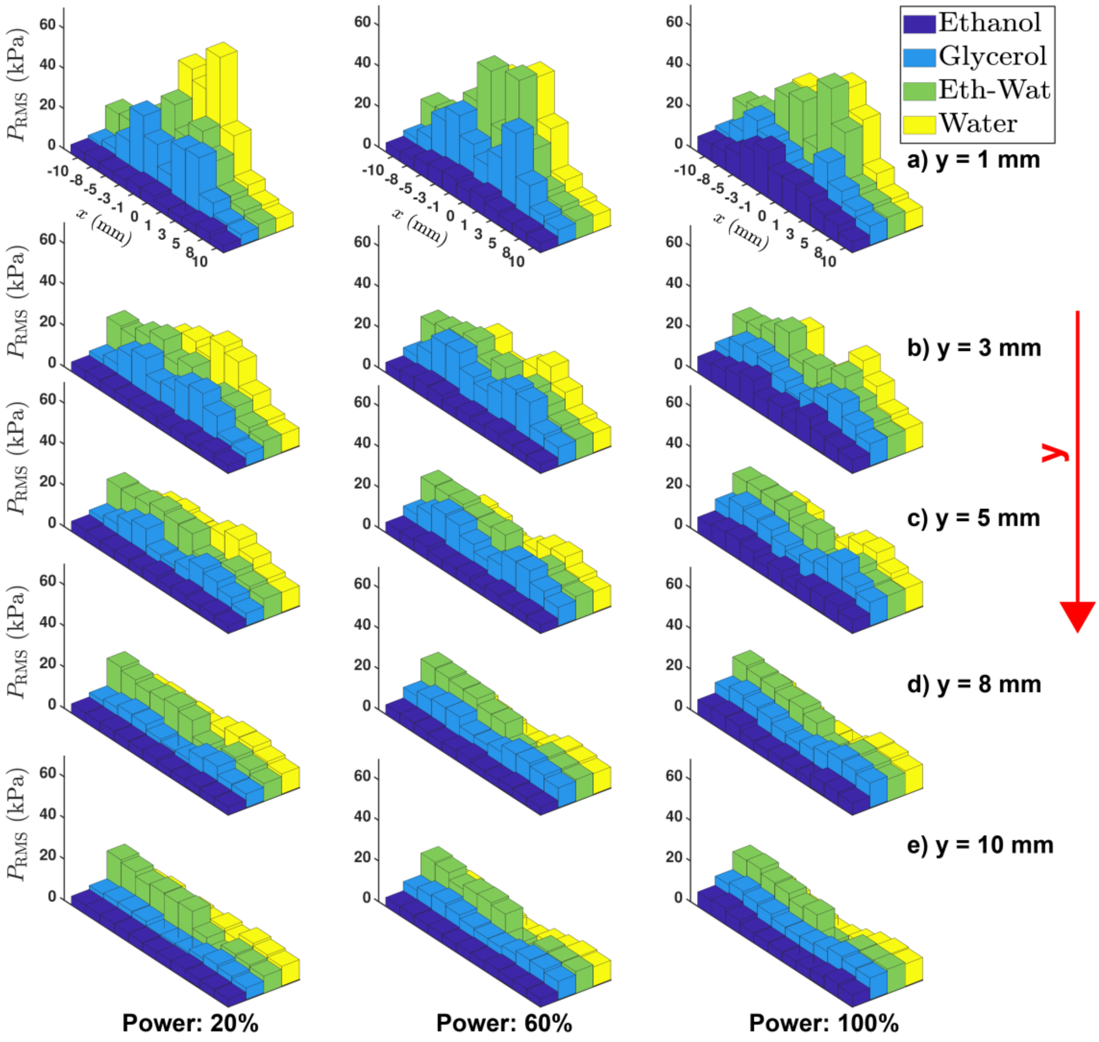

Figure A5. Comparison of the distribution of the RMS pressure (P_{RMS}) vs. horizontal position of the four working liquids for different vertical positions (y = 1-10 mm) and three input powers: 20% (left column), 60% (middle column) and 100% (right column).

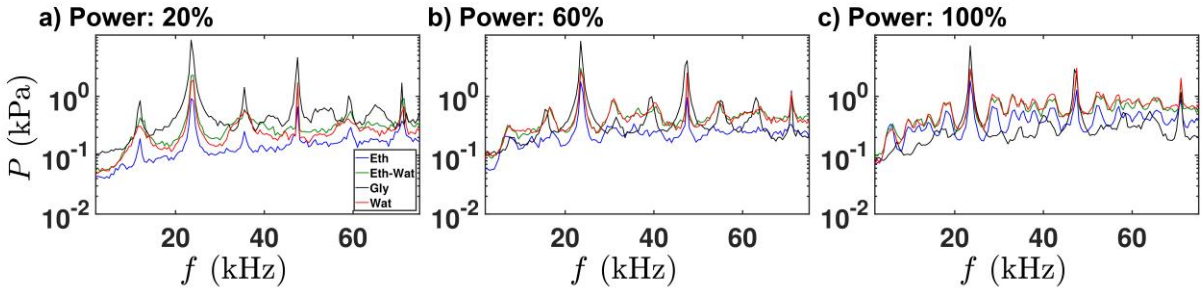

Figure A6. Log scale of the frequency response of acoustic emissions in kHz range for four working liquids at three input powers: a) 20%, b) 60% and 100%.

REFERENCES

1. Tzanakis, I., et al., *Characterizing the cavitation development and acoustic spectrum in various liquids.* Ultrasonics sonochemistry, 2017. **34**: p. 651-662.
2. James, G.S., *Lange's handbook of chemistry.* CD & W Inc, Wyoming, 2005.
3. Liu, C., E. Bonaccurso, and H.-J. Butt, *Evaporation of sessile water/ethanol drops in a controlled environment.* Physical Chemistry Chemical Physics, 2008. **10**(47): p. 7150-7157.
